# Supplementary material for: Mapping the evolution of fertility support policies in China: A content and instrumental analysis
Source: PLoS One. 2025 Oct 9;20(10):e0332137. doi: 10.1371/journal.pone.0332137 (PMC12510515; doi:10.1371/journal.pone.0332137)
Supplement: S1 Appendix — (ZIP) [file pone.0332137.s001.zip › S1 Appendix. 226 original policy documents/75-国务院关于落实《政府工作报告》重点工作分工的意见(FBM-CLI-2-5115313).doc]

国务院关于落实《政府工作报告》重点工作分工的意见

**发布部门：** [国务院](https://www.pkulaw.com/law/chl?Aggs.IssueDepartment=5)

**发文字号：**国发〔2022〕9号

**发布日期：**2022.03.21

**实施日期：**2022.03.21

**时效性：** [现行有效](https://www.pkulaw.com/law/chl?Aggs.TimelinessDic=01)

**效力级别：** [国务院规范性文件](https://www.pkulaw.com/law/chl?Aggs.EffectivenessDic=XC0203)

**法规类别：** [机关工作综合规定](https://www.pkulaw.com/law/chl?Aggs.Category=00301) [营商环境优化](https://dobusiness.pkulaw.com/)

国务院关于落实《政府工作报告》重点工作分工的意见
（国发〔2022〕9号）

各省、自治区、直辖市人民政府，国务院各部委、各直属机构：
　　为全面贯彻党的十九大和十九届历次全会精神，深入落实中央经济工作会议精神和十三届全国人大五次会议通过的《政府工作报告》部署，做好今年政府工作，实现经济社会发展目标任务，现就《政府工作报告》确定的重点工作，提出分工意见如下：
　　一、2022年经济社会发展总体要求和政策取向
　　（一）总体要求和主要预期目标。
　　**1．**要在以习近平同志为核心的党中央坚强领导下，以习近平新时代中国特色社会主义思想为指导，全面贯彻落实党的十九大和十九届历次全会精神，弘扬伟大建党精神，坚持稳中求进工作总基调，完整、准确、全面贯彻新发展理念，加快构建新发展格局，全面深化改革开放，坚持创新驱动发展，推动高质量发展，坚持以供给侧结构性改革为主线，统筹疫情防控和经济社会发展，统筹发展和安全，继续做好“六稳”、“六保”工作，持续改善民生，着力稳定宏观经济大盘，保持经济运行在合理区间，保持社会大局稳定，迎接党的二十大胜利召开。（国务院各部门分别负责，年内持续推进）

　　**2．**今年发展主要预期目标是：国内生产总值增长5.5%左右；城镇新增就业1100万人以上，城镇调查失业率全年控制在5.5%以内；居民消费价格涨幅3%左右；居民收入增长与经济增长基本同步；进出口保稳提质，国际收支基本平衡；粮食产量保持在1.3万亿斤以上；生态环境质量持续改善，主要污染物排放量继续下降；能耗强度目标在“十四五”规划期内统筹考核，并留有适当弹性，新增可再生能源和原料用能不纳入能源消费总量控制。（国家发展改革委、财政部、人力资源社会保障部、生态环境部、农业农村部、商务部、人民银行、海关总署、国家能源局、国家外汇局等按职责分工负责，12月底前完成）
　　（二）重大政策取向和要求。

　　**3．**完成今年发展目标任务，宏观政策要稳健有效，微观政策要持续激发市场主体活力，结构政策要着力畅通国民经济循环，科技政策要扎实落地，改革开放政策要激活发展动力，区域政策要增强发展的平衡性协调性，社会政策要兜住兜牢民生底线。各方面要围绕贯彻这些重大政策和要求，细化实化具体举措，形成推动发展的合力。（国务院各部门分别负责，年内持续推进）

　　**4．**要保持宏观政策连续性，强化跨周期和逆周期调节，增强有效性。积极的财政政策要提升效能，更加注重精准、可持续。稳健的货币政策要灵活适度，保持流动性合理充裕。就业优先政策要提质加力。政策发力适当靠前，及时动用储备政策工具，确保经济平稳运行。（国家发展改革委、财政部、人力资源社会保障部、人民银行等按职责分工负责，年内持续推进）

　　**5．**继续做好常态化疫情防控。坚持外防输入、内防反弹，不断优化完善防控措施，加强口岸城市疫情防控，加大对病毒变异的研究和防范力度，加快新型疫苗和特效药物研发，持续做好疫苗接种工作，更好发挥中医药独特作用，科学精准处置局部疫情，保持正常生产生活秩序。（国务院各部门分别负责，年内持续推进）

　　**6．**要坚持稳字当头、稳中求进。面对新的下行压力，要把稳增长放在更加突出的位置。各地区各部门要切实担负起稳定经济的责任，积极推出有利于经济稳定的政策。要统筹稳增长、调结构、推改革，加快转变发展方式，不搞粗放型发展。坚持实事求是，立足社会主义初级阶段基本国情，着力办好自己的事，尊重发展规律、客观实际和群众需求，因地制宜创造性开展工作，把各方面干事创业积极性充分调动起来。推动有效市场和有为政府更好结合，善于运用改革创新办法，激发市场活力和社会创造力。要坚持以人民为中心的发展思想，依靠共同奋斗，扎实推进共同富裕，不断实现人民对美好生活的向往。（国务院各部门分别负责，年内持续推进）
　　二、着力稳定宏观经济大盘，保持经济运行在合理区间
　　（三）提升积极的财政政策效能。

　　**7．**今年赤字率拟按2.8%左右安排、比去年有所下调，有利于增强财政可持续性。预计今年财政收入继续增长，加之特定国有金融机构和专营机构依法上缴近年结存的利润、调入预算稳定调节基金等，支出规模比去年扩大2万亿元以上，可用财力明显增加。新增财力要下沉基层，主要用于落实助企纾困、稳就业保民生政策，促进消费、扩大需求。今年安排中央本级支出增长3.9%，其中中央部门支出继续负增长。中央对地方转移支付增加约1.5万亿元、规模近9.8万亿元，增长18%、为多年来最大增幅。中央财政将更多资金纳入直达范围，省级财政也要加大对市县的支持，务必使基层落实惠企利民政策更有能力、更有动力。（财政部牵头，国家发展改革委、人力资源社会保障部、人民银行、税务总局、国家烟草局等按职责分工负责，12月底前完成）

　　**8．**要用好政府投资资金，带动扩大有效投资。今年拟安排地方政府专项债券3.65万亿元。强化绩效导向，坚持“资金、要素跟着项目走”，合理扩大使用范围，支持在建项目后续融资，开工一批具备条件的重大工程、新型基础设施、老旧公用设施改造等建设项目。民间投资在投资中占大头，要发挥重大项目牵引和政府投资撬动作用，完善相关支持政策，充分调动民间投资积极性。（国家发展改革委、财政部牵头，住房城乡建设部、自然资源部、生态环境部等按职责分工负责，年内持续推进）

　　**9．**要坚持政府过紧日子，更好节用裕民。大力优化支出结构，保障重点支出，严控一般性支出。盘活财政存量资金和闲置资产。各级政府必须艰苦奋斗、勤俭节约，中央政府和省级政府要带头。加强收支管理，严禁铺张浪费，不得违规新建楼堂馆所，不得搞形象工程，对违反财经纪律、肆意挥霍公款的要严查重处，一定要把宝贵资金用在发展紧要处、民生急需上。（财政部牵头，国家发展改革委、国管局等按职责分工负责，年内持续推进）
　　（四）加大稳健的货币政策实施力度。

　　**10．**发挥货币政策工具的总量和结构双重功能，为实体经济提供更有力支持。扩大新增贷款规模，保持货币供应量和社会融资规模增速与名义经济增速基本匹配，保持宏观杠杆率基本稳定。（人民银行牵头，年内持续推进）保持人民币汇率在合理均衡水平上的基本稳定。（人民银行、国家外汇局牵头，年内持续推进）进一步疏通货币政策传导机制，引导资金更多流向重点领域和薄弱环节，扩大普惠金融覆盖面。（人民银行、银保监会等按职责分工负责，年内持续推进）推动金融机构降低实际贷款利率、减少收费，让广大市场主体切身感受到融资便利度提升、综合融资成本实实在在下降。（人民银行、银保监会、证监会、国家外汇局、国家发展改革委等按职责分工负责，年内持续推进）
　　（五）强化就业优先政策。

　　**11．**大力拓宽就业渠道，注重通过稳市场主体来稳就业，增强创业带动就业作用。财税、金融等政策都要围绕就业优先实施，加大对企业稳岗扩岗的支持力度。（国家发展改革委、工业和信息化部、财政部、人力资源社会保障部、人民银行、税务总局、市场监管总局、银保监会、证监会等按职责分工负责，年内持续推进）各类专项促就业政策要强化优化，对就业创业的不合理限制要坚决清理取消。（人力资源社会保障部、国家发展改革委牵头，年内持续推进）各地都要千方百计稳定和扩大就业。（人力资源社会保障部牵头，年内持续推进）
　　（六）确保粮食能源安全。

　　**12．**保障粮食等重要农产品供应。（国家发展改革委、农业农村部、交通运输部、财政部、商务部、国家粮食和储备局等按职责分工负责，年内持续推进）继续做好能源、重要原材料保供稳价工作，保障民生和企业正常生产经营用电。（国家发展改革委、国家能源局牵头，年内持续推进）实施全面节约战略。增强国内资源生产保障能力，加快油气、矿产等资源勘探开发，完善国家战略物资储备制度，保障初级产品供给。（国家发展改革委、工业和信息化部、自然资源部、交通运输部、财政部、国家粮食和储备局、国家能源局等按职责分工负责，年内持续推进）打击哄抬物价等行为。保持物价水平基本稳定。（国家发展改革委、市场监管总局牵头，年内持续推进）
　　（七）防范化解重大风险。

　　**13．**继续按照稳定大局、统筹协调、分类施策、精准拆弹的基本方针，做好经济金融领域风险防范和处置工作。（国家发展改革委、财政部、人民银行、银保监会、证监会、国家外汇局等按职责分工负责，年内持续推进）压实地方属地责任、部门监管责任和企业主体责任，加强风险预警、防控机制和能力建设，设立金融稳定保障基金，发挥存款保险制度和行业保障基金的作用，运用市场化、法治化方式化解风险隐患，有效应对外部冲击，牢牢守住不发生系统性风险的底线。（人民银行牵头，国家发展改革委、司法部、财政部、银保监会、证监会、国家外汇局等按职责分工负责，9月底前完成金融稳定保障基金筹集相关工作，年内持续推进）
　　三、着力稳市场主体保就业，加大宏观政策实施力度
　　（八）实施新的组合式税费支持政策。

　　**14．**坚持阶段性措施和制度性安排相结合，减税与退税并举。一方面，延续实施扶持制造业、小微企业和个体工商户的减税降费政策，并提高减免幅度、扩大适用范围。对小规模纳税人阶段性免征增值税。对小微企业年应纳税所得额100万元至300万元部分，再减半征收企业所得税。各地也要结合实际，依法出台税费减免等有力措施，使减税降费力度只增不减，以稳定市场预期。另一方面，综合考虑为企业提供现金流支持、促进就业消费投资，大力改进因增值税税制设计类似于先缴后退的留抵退税制度，今年对留抵税额提前实行大规模退税。优先安排小微企业，对小微企业的存量留抵税额于6月底前一次性全部退还，增量留抵税额足额退还。重点支持制造业，全面解决制造业、科研和技术服务、生态环保、电力燃气、交通运输等行业留抵退税问题。增值税留抵退税力度显著加大，以有力提振市场信心。预计全年退税减税约2.5万亿元，其中留抵退税约1.5万亿元，退税资金全部直达企业。中央财政将加大对地方财力支持，补助资金直达市县，地方政府及有关部门要建立健全工作机制，加强资金调度，确保退税减税这项关键性举措落实到位，为企业雪中送炭，助企业焕发生机。（财政部、税务总局牵头，4月底前出台相关政策，年内持续推进）
　　（九）加强金融对实体经济的有效支持。

　　**15．**用好普惠小微贷款支持工具，增加支农支小再贷款，优化监管考核，推动普惠小微贷款明显增长、信用贷款和首贷户比重继续提升。（人民银行、银保监会等按职责分工负责，6月底前出台相关政策，年内持续推进）引导金融机构准确把握信贷政策，继续对受疫情影响严重的行业企业给予融资支持，避免出现行业性限贷、抽贷、断贷。对市场前景好的特殊困难行业企业给予“无缝续贷”。（人民银行、银保监会等按职责分工负责，年内持续推进）发挥好政策性、开发性金融作用。（财政部、人民银行、银保监会等按职责分工负责，6月底前出台分类分账改革相关方案，年内持续推进）推进涉企信用信息整合共享，加快税务、海关、电力等单位与金融机构信息联通，扩大政府性融资担保对小微企业的覆盖面，努力营造良好融资生态，进一步推动解决实体经济特别是中小微企业融资难题。（国家发展改革委、工业和信息化部、财政部、商务部、人民银行、海关总署、税务总局、市场监管总局、银保监会、国家能源局等按职责分工负责，年内持续推进）
　　（十）推动降低企业生产经营成本。

　　**16．**清理转供电环节不合理加价，支持地方对特殊困难行业用电实行阶段性优惠政策。（国家发展改革委、市场监管总局牵头，年内持续推进）引导大型平台企业降低收费，减轻中小商户负担。进一步清理规范行业协会商会、中介机构等收费。（国家发展改革委、市场监管总局、民政部等按职责分工负责，年内持续推进）要开展涉企违规收费专项整治行动，建立协同治理和联合惩戒机制，坚决查处乱收费、乱罚款、乱摊派。（国家发展改革委、工业和信息化部、财政部、市场监管总局牵头，有关部门按职责分工负责，12月底前完成）要加大拖欠中小企业账款清理力度，规范商业承兑汇票使用，机关、事业单位和国有企业要带头清欠。（工业和信息化部牵头，财政部、人民银行、国务院国资委等按职责分工负责，年内持续推进）餐饮、住宿、零售、文化、旅游、客运等行业就业容量大、受疫情影响重，各项帮扶政策都要予以倾斜，支持这些行业企业挺得住、过难关、有奔头。（国家发展改革委、财政部、交通运输部、住房城乡建设部、商务部、文化和旅游部、人民银行、税务总局、中国民航局、中国国家铁路集团有限公司等按职责分工负责，年内持续推进）
　　（十一）落实落细稳就业举措。

　　**17．**延续执行降低失业和工伤保险费率等阶段性稳就业政策。（人力资源社会保障部、财政部、税务总局牵头，4月底前出台相关政策，年内持续推进）对不裁员少裁员的企业，继续实施失业保险稳岗返还政策，明显提高中小微企业返还比例。（人力资源社会保障部、财政部牵头，4月底前出台相关政策，年内持续推进）今年高校毕业生超过1000万人，要加强就业创业指导、政策支持和不断线服务。（人力资源社会保障部、教育部、国家发展改革委、财政部等按职责分工负责，5月底前出台相关政策，年内持续推进）做好退役军人安置和就业保障，促进农民工就业，帮扶残疾人、零就业家庭成员就业。（人力资源社会保障部、国家发展改革委、财政部、农业农村部、退役军人部、国家乡村振兴局、中国残联等按职责分工负责，年内持续推进）深入开展大众创业万众创新，增强双创平台服务能力。（国家发展改革委牵头，年内持续推进）加强灵活就业服务，完善灵活就业社会保障政策，开展新就业形态职业伤害保障试点。（人力资源社会保障部、国家医保局、财政部、税务总局等按职责分工负责，年内持续推进）坚决防止和纠正性别、年龄、学历等就业歧视，大力营造公平就业环境。加强劳动保障监察执法，着力解决侵害劳动者合法权益的突出问题。（人力资源社会保障部、教育部、全国总工会、全国妇联等按职责分工负责，年内持续推进）增强公共就业服务针对性。（人力资源社会保障部牵头，年内持续推进）继续开展大规模职业技能培训，共建共享一批公共实训基地。（国家发展改革委、财政部、人力资源社会保障部等按职责分工负责，年内持续推进）使用1000亿元失业保险基金支持稳岗和培训，加快培养制造业高质量发展的急需人才，让更多劳动者掌握一技之长、让三百六十行行行人才辈出。（人力资源社会保障部、财政部牵头，4月底前出台相关政策，年内持续推进）
　　四、坚定不移深化改革，更大激发市场活力和发展内生动力
　　（十二）加快转变政府职能。

　　**18．**加强高标准市场体系建设，抓好要素市场化配置综合改革试点，加快建设全国统一大市场。继续扩大市场准入。（国家发展改革委牵头，有关部门按职责分工负责，年内持续推进）围绕打造市场化法治化国际化营商环境，持续推进“放管服”改革，对取消和下放审批事项要同步落实监管责任和措施。全面实行行政许可事项清单管理。（国务院办公厅牵头，有关部门按职责分工负责，年内持续推进）加强数字政府建设。（国务院办公厅牵头，国家发展改革委、科技部、工业和信息化部、财政部等按职责分工负责，年内持续推进）推动政务数据共享。（国务院办公厅牵头，有关部门按职责分工负责，年内持续推进）进一步压减各类证明事项。（国务院办公厅、司法部牵头，有关部门按职责分工负责，年内持续推进）扩大“跨省通办”范围，基本实现电子证照互通互认，便利企业跨区域经营，加快解决群众关切事项的异地办理问题。（国务院办公厅牵头，有关部门按职责分工负责，年内持续推进）推进居民身份证电子化，实现通过扫码办理需要用身份证的服务事项，同时保障好群众信息安全和隐私。（公安部、国务院办公厅牵头，有关部门按职责分工负责，年内持续推进）推进政务服务事项集成化办理。（国务院办公厅牵头，有关部门按职责分工负责，6月底前出台相关政策，年内持续推进）推出优化不动产登记、车辆检测等便民举措。（自然资源部、公安部、市场监管总局牵头，有关部门按职责分工负责，9月底前出台相关政策，年内持续推进）强化政府监管责任，严格落实行业主管部门、相关部门监管责任和地方政府属地监管责任，防止监管缺位。加快建立健全全方位、多层次、立体化监管体系，实现事前事中事后全链条全领域监管，提高监管效能。（国务院办公厅牵头，有关部门按职责分工负责，年内持续推进）抓紧完善重点领域、新兴领域、涉外领域监管规则，创新监管方法，提升监管精准性和有效性。（国务院办公厅牵头，国家发展改革委、工业和信息化部、生态环境部、商务部、应急部、人民银行、海关总署、市场监管总局、银保监会、证监会、国家药监局等按职责分工负责，年内持续推进）深入推进公平竞争政策实施，加强反垄断和反不正当竞争，维护公平有序的市场环境。（市场监管总局牵头，国家发展改革委、科技部、工业和信息化部、财政部、商务部、人民银行、银保监会、证监会、全国工商联等按职责分工负责，年内持续推进）
　　（十三）促进多种所有制经济共同发展。

　　**19．**坚持和完善社会主义基本经济制度，坚持“两个毫不动摇”。要正确认识和把握资本的特性和行为规律，支持和引导资本规范健康发展。依法平等保护企业产权、自主经营权和企业家合法权益，营造各类所有制企业竞相发展的良好环境。（国家发展改革委、工业和信息化部、商务部、人民银行、国务院国资委、市场监管总局、银保监会、证监会、全国工商联等按职责分工负责，年内持续推进）完成国企改革三年行动任务，加快国有经济布局优化和结构调整，深化混合所有制改革，加强国有资产监管，促进国企聚焦主责主业、提升产业链供应链支撑和带动能力。（国务院国资委、国家发展改革委、财政部等按职责分工负责，年内持续推进）落实支持民营经济发展的政策措施，鼓励引导民营企业改革创新，构建亲清政商关系。弘扬企业家精神，制定涉企政策要多听市场主体意见，尊重市场规律，支持企业家专注创业创新、安心经营发展。（国家发展改革委、工业和信息化部、国务院国资委、全国工商联、中国贸促会等按职责分工负责，年内持续推进）
　　（十四）推进财税金融体制改革。

　　**20．**深化预算绩效管理改革，增强预算的约束力和透明度。（财政部牵头，年内持续推进）推进省以下财政体制改革。（财政部牵头，9月底前出台相关政策，年内持续推进）完善税收征管制度，依法打击偷税骗税。（税务总局牵头，9月底前出台相关政策，年内持续推进）加强和改进金融监管。（人民银行、银保监会、证监会、国家外汇局等按职责分工负责，年内持续推进）深化中小银行股权结构和公司治理改革，加快不良资产处置。（银保监会牵头，年内持续推进）完善民营企业债券融资支持机制，全面实行股票发行注册制，促进资本市场平稳健康发展。（人民银行、国家发展改革委、证监会等按职责分工负责，年内持续推进）
　　五、深入实施创新驱动发展战略，巩固壮大实体经济根基
　　（十五）提升科技创新能力。

　　**21．**实施基础研究十年规划，加强长期稳定支持，提高基础研究经费占全社会研发经费比重。（科技部、财政部、教育部、国务院国资委、中科院、工程院、国家自然科学基金委等按职责分工负责，年内持续推进）实施科技体制改革三年攻坚方案，强化国家战略科技力量，加强国家实验室和全国重点实验室建设，发挥好高校和科研院所作用，改进重大科技项目立项和管理方式，深化科技评价激励制度改革。（科技部、国家发展改革委、财政部牵头，国家国防科工局等按职责分工负责，年内持续推进）支持各地加大科技投入，开展各具特色的区域创新。（科技部、国家发展改革委牵头，有关部门按职责分工负责，年内持续推进）加强科普工作。推进国际科技合作。（科技部牵头，外交部、国家发展改革委、中科院、工程院、中国科协、国家自然科学基金委等按职责分工负责，年内持续推进）加快建设世界重要人才中心和创新高地，完善人才发展体制机制，弘扬科学家精神，加大对青年科研人员支持力度，让各类人才潜心钻研、尽展其能。（科技部、教育部、中国科协等按职责分工负责，年内持续推进）
　　（十六）加大企业创新激励力度。

　　**22．**强化企业创新主体地位，持续推进关键核心技术攻关，深化产学研用结合，促进科技成果转移转化。（科技部、国家发展改革委、工业和信息化部、教育部、财政部、农业农村部、国务院国资委、国家国防科工局牵头，有关部门按职责分工负责，年内持续推进）加强知识产权保护和运用。（国家知识产权局牵头，教育部、科技部、工业和信息化部、司法部、市场监管总局等按职责分工负责，年内持续推进）促进创业投资发展，创新科技金融产品和服务，提升科技中介服务专业化水平。（人民银行、银保监会、证监会、科技部、国家发展改革委、税务总局等按职责分工负责，6月底前出台相关政策，年内持续推进）加大研发费用加计扣除政策实施力度，将科技型中小企业加计扣除比例从75%提高到100%，对企业投入基础研究实行税收优惠，完善设备器具加速折旧、高新技术企业所得税优惠等政策，这相当于国家对企业创新给予大规模资金支持。（财政部、税务总局、科技部牵头，9月底前出台相关政策，年内持续推进）要落实好各类创新激励政策，以促进企业加大研发投入，培育壮大新动能。（科技部、国家发展改革委、财政部、税务总局、国务院国资委牵头，年内持续推进）
　　（十七）增强制造业核心竞争力。

　　**23．**促进工业经济平稳运行，加强原材料、关键零部件等供给保障，实施龙头企业保链稳链工程，维护产业链供应链安全稳定。（国家发展改革委、工业和信息化部牵头，年内持续推进）引导金融机构增加制造业中长期贷款。（人民银行、银保监会牵头，年内持续推进）启动一批产业基础再造工程项目，促进传统产业升级，大力推进智能制造，加快发展先进制造业集群，实施国家战略性新兴产业集群工程。（工业和信息化部、国家发展改革委牵头，年内持续推进）着力培育“专精特新”企业，在资金、人才、孵化平台搭建等方面给予大力支持。（工业和信息化部牵头，财政部、人力资源社会保障部、人民银行、银保监会、证监会等按职责分工负责，年内持续推进）推进质量强国建设，推动产业向中高端迈进。（市场监管总局、国家发展改革委、科技部、工业和信息化部等按职责分工负责，年内持续推进）
　　（十八）促进数字经济发展。

　　**24．**加强数字中国建设整体布局。建设数字信息基础设施，逐步构建全国一体化大数据中心体系，推进5G规模化应用，促进产业数字化转型。（国家发展改革委、工业和信息化部牵头，年内持续推进）发展智慧城市、数字乡村。（国家发展改革委、工业和信息化部、住房城乡建设部、农业农村部等按职责分工负责，年内持续推进）加快发展工业互联网，培育壮大集成电路、人工智能等数字产业，提升关键软硬件技术创新和供给能力。（国家发展改革委、工业和信息化部、科技部等按职责分工负责，年内持续推进）完善数字经济治理，培育数据要素市场，释放数据要素潜力，提高应用能力，更好赋能经济发展、丰富人民生活。（国家发展改革委、工业和信息化部、商务部、市场监管总局等按职责分工负责，年内持续推进）
　　六、坚定实施扩大内需战略，推进区域协调发展和新型城镇化
　　（十九）推动消费持续恢复。

　　**25．**多渠道促进居民增收，完善收入分配制度，提升消费能力。（国家发展改革委牵头，财政部、人力资源社会保障部等按职责分工负责，年内持续推进）推动线上线下消费深度融合，促进生活服务消费恢复，发展消费新业态新模式。（商务部、国家发展改革委牵头，年内持续推进）继续支持新能源汽车消费，鼓励地方开展绿色智能家电下乡和以旧换新。（国家发展改革委、工业和信息化部、财政部、生态环境部、住房城乡建设部、商务部、国家能源局等按职责分工负责，年内持续推进）加大社区养老、托幼等配套设施建设力度，在规划、用地、用房等方面给予更多支持。（住房城乡建设部、民政部、国家发展改革委、自然资源部、国家卫生健康委等按职责分工负责，年内持续推进）促进家政服务业提质扩容。（国家发展改革委、商务部牵头，年内持续推进）加强县域商业体系建设，发展农村电商和快递物流配送。（商务部、国家邮政局牵头，农业农村部、交通运输部、供销合作总社、中国邮政集团有限公司等按职责分工负责，年内持续推进）提高产品和服务质量，强化消费者权益保护，着力适应群众需求、增强消费意愿。（市场监管总局牵头，国家发展改革委、科技部、工业和信息化部、农业农村部等按职责分工负责，年内持续推进）
　　（二十）积极扩大有效投资。

　　**26．**围绕国家重大战略部署和“十四五”规划，适度超前开展基础设施投资。建设重点水利工程、综合立体交通网、重要能源基地和设施，加快城市燃气管道、给排水管道等管网更新改造，完善防洪排涝设施，继续推进地下综合管廊建设。中央预算内投资安排6400亿元。政府投资更多向民生项目倾斜，加大社会民生领域补短板力度。（国家发展改革委、工业和信息化部、民政部、财政部、住房城乡建设部、交通运输部、水利部、农业农村部、国家卫生健康委、应急部、国家能源局、中国民航局、中国国家铁路集团有限公司等按职责分工负责，年内持续推进）深化投资审批制度改革，做好用地、用能等要素保障，对国家重大项目要实行能耗单列。要优化投资结构，破解投资难题，切实把投资关键作用发挥出来。（国家发展改革委、财政部、自然资源部、生态环境部、人民银行、银保监会、国家能源局等按职责分工负责，年内持续推进）
　　（二十一）增强区域发展平衡性协调性。

　　**27．**深入实施区域重大战略和区域协调发展战略。推进京津冀协同发展、长江经济带发展、粤港澳大湾区建设、长三角一体化发展、黄河流域生态保护和高质量发展，高标准高质量建设雄安新区，支持北京城市副中心建设。推动西部大开发形成新格局，推动东北振兴取得新突破，推动中部地区高质量发展，鼓励东部地区加快推进现代化，支持产业梯度转移和区域合作。支持革命老区、民族地区、边疆地区加快发展。发展海洋经济，建设海洋强国。经济大省要充分发挥优势，增强对全国发展的带动作用。经济困难地区要用好国家支持政策，挖掘自身潜力，努力促进经济恢复发展。（国家发展改革委牵头，年内持续推进）
　　（二十二）提升新型城镇化质量。

　　**28．**有序推进城市更新，加强市政设施和防灾减灾能力建设，开展老旧建筑和设施安全隐患排查整治，再开工改造一批城镇老旧小区，支持加装电梯等设施，推进无障碍环境建设和公共设施适老化改造。（住房城乡建设部牵头，国家发展改革委、财政部、自然资源部、应急部、中国残联等按职责分工负责，年内持续推进）健全常住地提供基本公共服务制度。（国家发展改革委、财政部、教育部、公安部、民政部、人力资源社会保障部、住房城乡建设部、国家卫生健康委、退役军人部、国家医保局等按职责分工负责，年内持续推进）加强县城基础设施建设。稳步推进城市群、都市圈建设，促进大中小城市和小城镇协调发展。（国家发展改革委、住房城乡建设部牵头，年内持续推进）推进成渝地区双城经济圈建设。（国家发展改革委牵头，有关部门与重庆市人民政府、四川省人民政府等按职责分工负责，年内持续推进）严控撤县建市设区。（民政部牵头，有关部门按职责分工负责，年内持续推进）在城乡规划建设中做好历史文化保护传承，节约集约用地。（自然资源部、住房城乡建设部、文化和旅游部、国家文物局等按职责分工负责，年内持续推进）要深入推进以人为核心的新型城镇化，不断提高人民生活质量。（国家发展改革委牵头，年内持续推进）
　　七、大力抓好农业生产，促进乡村全面振兴
　　（二十三）加强粮食等重要农产品稳产保供。

　　**29．**稳定粮食播种面积，优化粮食结构，针对小麦晚播强化夏粮田间管理，促进大豆和油料增产。（农业农村部、国家林草局等按职责分工负责，年内持续推进）适当提高稻谷、小麦最低收购价。（国家发展改革委牵头，财政部、农业农村部、国家粮食和储备局等按职责分工负责，4月底前完成）保障化肥等农资供应和价格稳定，给种粮农民再次发放农资补贴，加大对主产区支持力度，让农民种粮有合理收益、主产区抓粮有内在动力。（国家发展改革委、财政部、农业农村部、工业和信息化部、生态环境部、交通运输部、商务部、市场监管总局、供销合作总社等按职责分工负责，年内持续推进）坚决守住18亿亩耕地红线，划足划实永久基本农田，切实遏制耕地“非农化”、防止“非粮化”。（自然资源部、农业农村部等按职责分工负责，年内持续推进）加强中低产田改造，新建1亿亩高标准农田，新建改造一批大中型灌区。（农业农村部、水利部牵头，年内持续推进）加大黑土地保护和盐碱地综合利用力度。（国家发展改革委、农业农村部、财政部、水利部、科技部、中科院、国家林草局、自然资源部等按职责分工负责，年内持续推进）支持黄河流域发展节水农业、旱作农业。（农业农村部、水利部、国家发展改革委、财政部等按职责分工负责，年内持续推进）启动第三次全国土壤普查。（农业农村部牵头，自然资源部、国家发展改革委、财政部、生态环境部、水利部、国家统计局、中科院、国家林草局等按职责分工负责，年内持续推进）加快推进种业振兴，加强农业科技攻关和推广应用，提高农机装备水平。（农业农村部、科技部、国家发展改革委、工业和信息化部、国家林草局等按职责分工负责，年内持续推进）提升农业气象灾害防控和动植物疫病防治能力。（农业农村部牵头，中国气象局、海关总署、国家林草局等按职责分工负责，年内持续推进）加强生猪产能调控，抓好畜禽、水产、蔬菜等生产供应，加快发展现代化设施种养业。支持棉花、甘蔗等生产。（农业农村部牵头，年内持续推进）保障国家粮食安全各地区都有责任，粮食调入地区更要稳定粮食生产。各方面要共同努力，装满“米袋子”、充实“菜篮子”，把14亿多中国人的饭碗牢牢端在自己手中。（国家发展改革委、农业农村部、财政部、商务部、国家粮食和储备局等按职责分工负责，年内持续推进）
　　（二十四）全面巩固拓展脱贫攻坚成果。

　　**30．**完善落实防止返贫监测帮扶机制，确保不发生规模性返贫。（国家乡村振兴局牵头，年内持续推进）支持脱贫地区发展特色产业，加强劳务协作、职业技能培训，促进脱贫人口持续增收。（农业农村部、国家乡村振兴局、人力资源社会保障部、全国工商联等按职责分工负责，年内持续推进）强化国家乡村振兴重点帮扶县帮扶措施，做好易地搬迁后续扶持，深化东西部协作、定点帮扶和社会力量帮扶，大力实施“万企兴万村”行动，增强脱贫地区自我发展能力。（国家乡村振兴局、国家发展改革委、农业农村部、全国工商联等按职责分工负责，年内持续推进）
　　（二十五）扎实稳妥推进农村改革发展。

　　**31．**开展好第二轮土地承包到期后再延长30年整县试点。（农业农村部牵头，年内持续推进）深化供销社、集体产权、集体林权、国有林区林场、农垦等改革。积极发展新型农村集体经济。（国家发展改革委、自然资源部、农业农村部、国家林草局、供销合作总社等按职责分工负责，年内持续推进）加强农村金融服务，加快发展乡村产业。（人民银行、银保监会、证监会、国家外汇局、农业农村部、国家乡村振兴局、国家发展改革委、财政部、农业发展银行等按职责分工负责，年内持续推进）壮大县域经济。（国家发展改革委、工业和信息化部、农业农村部、商务部、国家乡村振兴局等按职责分工负责，年内持续推进）严格规范村庄撤并，保护传统村落和乡村风貌。启动乡村建设行动，强化规划引领，加强水电路气信邮等基础设施建设，因地制宜推进农村改厕和污水垃圾处理。（农业农村部、国家乡村振兴局、国家发展改革委、工业和信息化部、民政部、财政部、自然资源部、生态环境部、住房城乡建设部、交通运输部、水利部、文化和旅游部、国家卫生健康委、国家邮政局、国家文物局等按职责分工负责，年内持续推进）深入开展文明村镇建设。（有关部门按职责分工负责，年内持续推进）强化农民工工资拖欠治理，支持农民工就业创业，一定要让广大农民有更多务工增收的渠道。（人力资源社会保障部、住房城乡建设部、交通运输部、水利部、农业农村部、国家乡村振兴局等按职责分工负责，年内持续推进）
　　八、扩大高水平对外开放，推动外贸外资平稳发展
　　（二十六）多措并举稳定外贸。

　　**32．**扩大出口信用保险对中小微外贸企业的覆盖面，加强出口信贷支持，优化外汇服务，加快出口退税进度，帮助外贸企业稳订单稳生产。（商务部、财政部、人民银行、税务总局、银保监会、国家外汇局、进出口银行、中国出口信用保险公司、中国贸促会等按职责分工负责，年内持续推进）加快发展外贸新业态新模式，充分发挥跨境电商作用，支持建设一批海外仓。（商务部牵头，年内持续推进）积极扩大优质产品和服务进口。（商务部、财政部、海关总署牵头，年内持续推进）创新发展服务贸易、数字贸易，推进实施跨境服务贸易负面清单。（商务部牵头，年内持续推进）深化通关便利化改革，加快国际物流体系建设，助力外贸降成本、提效率。（海关总署、商务部、交通运输部、国家发展改革委、国家移民局、中国贸促会、中国国家铁路集团有限公司等按职责分工负责，年内持续推进）
　　（二十七）积极利用外资。

　　**33．**深入实施外资准入负面清单，落实好外资企业国民待遇。扩大鼓励外商投资范围，支持外资加大中高端制造、研发、现代服务等领域和中西部、东北地区投资。优化外资促进服务，推动重大项目加快落地。（国家发展改革委、商务部等按职责分工负责，年内持续推进）扎实推进自贸试验区、海南自由贸易港建设，推动开发区改革创新，提高综合保税区发展水平，增设服务业扩大开放综合试点。（国家发展改革委、商务部、科技部、海关总署与海南省人民政府等按职责分工负责，年内持续推进）开放的中国大市场，必将为各国企业在华发展提供更多机遇。（国家发展改革委、商务部等按职责分工负责，年内持续推进）
　　（二十八）高质量共建“一带一路”。

　　**34．**坚持共商共建共享，巩固互联互通合作基础，稳步拓展合作新领域。推进西部陆海新通道建设。有序开展对外投资合作，有效防范海外风险。（国家发展改革委、商务部、外交部牵头，有关部门按职责分工负责，年内持续推进）
　　（二十九）深化多双边经贸合作。

　　**35．**区域全面经济伙伴关系协定形成了全球最大自由贸易区，要支持企业用好优惠关税、原产地累积等规则，扩大贸易和投资合作。（商务部、海关总署牵头，年内持续推进）推动与更多国家和地区商签高标准自贸协定。坚定维护多边贸易体制，积极参与世贸组织改革。中国愿与世界各国加强互利合作，实现共赢多赢。（商务部牵头，年内持续推进）
　　九、持续改善生态环境，推动绿色低碳发展
　　（三十）加强生态环境综合治理。

　　**36．**深入打好污染防治攻坚战。强化大气多污染物协同控制和区域协同治理，加大重要河湖、海湾污染整治力度，持续推进土壤污染防治。（生态环境部、国家发展改革委、工业和信息化部、财政部、自然资源部、住房城乡建设部、交通运输部、水利部、农业农村部、公安部、应急部、市场监管总局、国家能源局等按职责分工负责，年内持续推进）加强固体废物和新污染物治理，推行垃圾分类和减量化、资源化。（生态环境部、住房城乡建设部、国家发展改革委、工业和信息化部、农业农村部、国家卫生健康委、商务部、市场监管总局等按职责分工负责，年内持续推进）完善节能节水、废旧物资循环利用等环保产业支持政策。（国家发展改革委、住房城乡建设部、水利部、商务部等按职责分工负责，年内持续推进）加强生态环境分区管控，科学开展国土绿化，统筹山水林田湖草沙系统治理，保护生物多样性，推进以国家公园为主体的自然保护地体系建设，要让我们生活的家园更绿更美。（生态环境部、自然资源部、国家发展改革委、财政部、住房城乡建设部、水利部、农业农村部、国家林草局等按职责分工负责，年内持续推进）
　　（三十一）有序推进碳达峰碳中和工作。

　　**37．**落实碳达峰行动方案。（国家发展改革委牵头，有关部门按职责分工负责，年内持续推进）推动能源革命，确保能源供应，立足资源禀赋，坚持先立后破、通盘谋划，推进能源低碳转型。加强煤炭清洁高效利用，有序减量替代，推动煤电节能降碳改造、灵活性改造、供热改造。推进大型风光电基地及其配套调节性电源规划建设，加强抽水蓄能电站建设，提升电网对可再生能源发电的消纳能力。支持生物质能发展。（国家发展改革委、科技部、工业和信息化部、国家能源局等按职责分工负责，年内持续推进）推进绿色低碳技术研发和推广应用，建设绿色制造和服务体系，推进钢铁、有色、石化、化工、建材等行业节能降碳，强化交通和建筑节能。坚决遏制高耗能、高排放、低水平项目盲目发展。（国家发展改革委牵头，科技部、工业和信息化部、生态环境部、住房城乡建设部、交通运输部等按职责分工负责，年内持续推进）提升生态系统碳汇能力。推动能耗“双控”向碳排放总量和强度“双控”转变，完善减污降碳激励约束政策，发展绿色金融，加快形成绿色低碳生产生活方式。（国家发展改革委、自然资源部、生态环境部、人民银行、国家统计局、国家林草局等按职责分工负责，年内持续推进）
　　十、切实保障和改善民生，加强和创新社会治理
　　（三十二）促进教育公平与质量提升。

　　**38．**落实立德树人根本任务。推动义务教育优质均衡发展和城乡一体化，依据常住人口规模配置教育资源，保障适龄儿童就近入学，解决好进城务工人员子女就学问题。加强乡村教师定向培养、在职培训与待遇保障。继续做好义务教育阶段减负工作。多渠道增加普惠性学前教育资源。加强县域普通高中建设。办好特殊教育、继续教育、专门教育，支持和规范民办教育发展。提升国家通用语言文字普及程度和质量。发展现代职业教育，改善职业教育办学条件，完善产教融合办学体制，增强职业教育适应性。推进高等教育内涵式发展，优化高等教育布局，分类建设一流大学和一流学科，加快培养理工农医类专业紧缺人才，支持中西部高等教育发展。高校招生继续加大对中西部和农村地区倾斜力度。加强师德师风建设。健全学校家庭社会协同育人机制。发展在线教育。完善终身学习体系。倡导全社会尊师重教。我国有2.9亿在校学生，要坚持把教育这个关乎千家万户和中华民族未来的大事办好。（教育部牵头，国家发展改革委、科技部、工业和信息化部、国家民委、民政部、财政部、人力资源社会保障部、自然资源部、住房城乡建设部、农业农村部、国家卫生健康委、市场监管总局、体育总局、国家疾控局、银保监会、全国妇联、国务院妇儿工委办公室、中国残联等按职责分工负责，年内持续推进）全面落实义务教育教师工资待遇。（人力资源社会保障部、教育部、财政部牵头，年内持续推进）进一步加大向农村和边远地区义务教育的投入力度。（教育部牵头，财政部、国家发展改革委等按职责分工负责，年内持续推进）
　　（三十三）提高医疗卫生服务能力。

　　**39．**居民医保和基本公共卫生服务经费人均财政补助标准分别再提高30元和5元。（财政部、国家卫生健康委、国家医保局、国家疾控局牵头，6月底前出台相关政策，年内持续推进）推动基本医保省级统筹。（国家医保局、财政部、税务总局牵头，年内持续推进）推进药品和高值医用耗材集中带量采购，确保生产供应。（国家医保局、工业和信息化部、财政部等按职责分工负责，年内持续推进）强化药品疫苗质量安全监管。（国家药监局牵头，工业和信息化部、公安部、商务部、国家卫生健康委、市场监管总局、国家医保局、国家中医药局、国家疾控局等按职责分工负责，年内持续推进）深化医保支付方式改革，加强医保基金监管。（国家医保局牵头，财政部、国家卫生健康委等按职责分工负责，年内持续推进）完善跨省异地就医直接结算办法。（国家医保局牵头，财政部等按职责分工负责，6月底前出台相关政策，年内持续推进）实现全国医保用药范围基本统一。（国家医保局牵头，12月底前完成）

　　**40．**坚持预防为主，加强健康教育和健康管理，深入推进健康中国行动。（国家卫生健康委、教育部、体育总局牵头，国家发展改革委、民政部、生态环境部、住房城乡建设部、农业农村部、市场监管总局、国家中医药局、国家疾控局等按职责分工负责，年内持续推进）逐步提高心脑血管病、癌症等慢性病和肺结核、肝炎等传染病防治服务保障水平，加强罕见病研究和用药保障。（工业和信息化部、国家卫生健康委、科技部、国家医保局、国家药监局等按职责分工负责，年内持续推进）健全疾病预防控制网络，促进医防协同，加强公共卫生队伍建设，提高重大疫情监测预警、流调溯源和应急处置能力。（国家卫生健康委、国家疾控局、公安部、工业和信息化部、国家发展改革委、教育部、科技部、财政部、人力资源社会保障部、交通运输部、商务部、市场监管总局、海关总署、国家移民局、中国民航局、国家中医药局、国家药监局等按职责分工负责，年内持续推进）推动公立医院综合改革和高质量发展。规范医疗机构收费和服务，继续帮扶因疫情遇困的医疗机构，补齐妇幼儿科、精神卫生、老年医学等服务短板。（国家卫生健康委、国家发展改革委、市场监管总局、财政部、教育部、人力资源社会保障部、民政部、国务院国资委、国家医保局、国家中医药局、国家疾控局等按职责分工负责，年内持续推进）坚持中西医并重，加大中医药振兴发展支持力度，推进中医药综合改革。（国家中医药局、国家卫生健康委、国家发展改革委牵头，教育部、科技部、工业和信息化部、财政部、人力资源社会保障部、农业农村部、国家医保局、国家林草局、国家药监局、国家知识产权局等按职责分工负责，年内持续推进）落实和完善乡村医生待遇保障与激励政策。持续推进分级诊疗和优化就医秩序，加快建设国家、省级区域医疗中心，推动优质医疗资源向市县延伸，提升基层防病治病能力，使群众就近得到更好医疗卫生服务。（国家卫生健康委、国家发展改革委、财政部、教育部、人力资源社会保障部、国家中医药局、国家疾控局、国家医保局等按职责分工负责，年内持续推进）
　　（三十四）加强社会保障和服务。

　　**41．**稳步实施企业职工基本养老保险全国统筹。（人力资源社会保障部、财政部、税务总局牵头，年内持续推进）适当提高退休人员基本养老金和城乡居民基础养老金标准，确保按时足额发放。（人力资源社会保障部、财政部牵头，6月底前出台相关政策，年内持续推进）继续规范发展第三支柱养老保险。（人力资源社会保障部、财政部、银保监会牵头，4月底前出台相关政策，年内持续推进）加快推进工伤和失业保险省级统筹。（人力资源社会保障部、财政部、税务总局牵头，6月底前出台相关政策，年内持续推进）做好军人军属、退役军人和其他优抚对象优待抚恤工作。（退役军人部牵头，财政部等按职责分工负责，年内持续推进）积极应对人口老龄化，加快构建居家社区机构相协调、医养康养相结合的养老服务体系。优化城乡养老服务供给，支持社会力量提供日间照料、助餐助洁、康复护理等服务，稳步推进长期护理保险制度试点，鼓励发展农村互助式养老服务，创新发展老年教育，推动老龄事业和产业高质量发展。（国家发展改革委、民政部、国家卫生健康委、教育部、财政部、人力资源社会保障部、自然资源部、住房城乡建设部、商务部、农业农村部、市场监管总局、国家医保局、银保监会、国家中医药局、国家乡村振兴局、全国老龄办、中国残联等按职责分工负责，年内持续推进）完善三孩生育政策配套措施，多渠道发展普惠托育服务，减轻家庭生育、养育、教育负担。（国家卫生健康委、国家发展改革委、财政部、人力资源社会保障部、教育部、商务部、住房城乡建设部、自然资源部、国家医保局等按职责分工负责，年内持续推进）将3岁以下婴幼儿照护费用纳入个人所得税专项附加扣除。（财政部、税务总局牵头，公安部、民政部、国家卫生健康委等按职责分工负责，3月底前出台相关政策，年内持续推进）强化未成年人保护和心理健康教育。（民政部牵头，有关部门按职责分工负责，年内持续推进）提升残疾预防和康复服务水平。（民政部、国家卫生健康委、中国残联牵头，有关部门按职责分工负责，年内持续推进）加强民生兜底保障和遇困群众救助，努力做到应保尽保、应助尽助。（民政部、国家卫生健康委、应急部、国家医保局牵头，年内持续推进）
　　（三十五）继续保障好群众住房需求。

　　**42．**坚持房子是用来住的、不是用来炒的定位，探索新的发展模式，坚持租购并举，加快发展长租房市场，推进保障性住房建设，支持商品房市场更好满足购房者的合理住房需求，稳地价、稳房价、稳预期，因城施策促进房地产业良性循环和健康发展。（住房城乡建设部牵头，国家发展改革委、财政部、自然资源部、人民银行、税务总局、银保监会、证监会等按职责分工负责，年内持续推进）
　　（三十六）丰富人民群众精神文化生活。

　　**43．**培育和践行社会主义核心价值观，深化群众性精神文明创建。繁荣新闻出版、广播影视、文学艺术、哲学社会科学和档案等事业。深入推进全民阅读。加强和创新互联网内容建设，深化网络生态治理。推进公共文化数字化建设，促进基层文化设施布局优化和资源共享，扩大优质文化产品和服务供给，支持文化产业发展。传承弘扬中华优秀传统文化，加强文物古籍保护利用和非物质文化遗产保护传承，推进国家文化公园建设。（文化和旅游部、国家发展改革委、广电总局、教育部、社科院、国家文物局等按职责分工负责，年内持续推进）用好北京冬奥会、冬残奥会遗产，发展冰雪运动和冰雪产业。建设群众身边的体育场地设施，促进全民健身蔚然成风。（体育总局、国家发展改革委、住房城乡建设部与北京市人民政府、河北省人民政府等按职责分工负责，年内持续推进）
　　（三十七）推进社会治理共建共治共享。

　　**44．**促进人民安居乐业、社会安定有序。创新和完善基层社会治理，强化社区服务功能，加强社会动员体系建设，提升基层治理能力。（民政部、工业和信息化部、公安部、司法部、住房城乡建设部、农业农村部、国家卫生健康委、国家乡村振兴局等按职责分工负责，年内持续推进）健全社会信用体系。（国家发展改革委、人民银行牵头，有关部门按职责分工负责，6月底前出台相关政策，年内持续推进）发展社会工作，支持社会组织、人道救助、志愿服务、公益慈善等健康发展。健全老年人、残疾人关爱服务体系。（民政部、国务院妇儿工委办公室、共青团中央、全国老龄办、全国妇联、中国残联、中国红十字会总会等按职责分工负责，年内持续推进）严厉打击拐卖、收买妇女儿童犯罪行为，坚决保障妇女儿童合法权益。（公安部、民政部、司法部、国务院妇儿工委办公室、全国妇联、共青团中央等按职责分工负责，年内持续推进）完善信访制度，加强矛盾纠纷排查化解，依法及时解决群众合理诉求。（国家信访局牵头，中央信访工作联席会议成员单位和有关部门按职责分工负责，年内持续推进）重视社会心理服务。（有关部门按职责分工负责，年内持续推进）强化公共法律服务和法律援助。（司法部牵头，年内持续推进）

　　**45．**提高防灾减灾救灾和应急救援能力，做好洪涝干旱、森林草原火灾、地质灾害、地震等防御和气象服务。（应急部、自然资源部、住房城乡建设部、水利部、工业和信息化部、中国气象局、国家粮食和储备局、国家林草局、中国地震局等按职责分工负责，年内持续推进）严格食品全链条质量安全监管。（市场监管总局、公安部、农业农村部、国家卫生健康委、海关总署、教育部、生态环境部、国家粮食和储备局、工业和信息化部等按职责分工负责，年内持续推进）落实安全生产责任和管理制度，深入开展安全生产专项整治三年行动，有效遏制重特大事故发生。（应急部牵头，工业和信息化部、公安部、自然资源部、住房城乡建设部、交通运输部、农业农村部、生态环境部、国家能源局、国家矿山安监局等按职责分工负责，年内持续推进）推进国家安全体系和能力建设。（安全部等按职责分工负责，年内持续推进）强化网络安全、数据安全和个人信息保护。（有关部门按职责分工负责，年内持续推进）加强社会治安综合治理，推动扫黑除恶常态化，坚决防范和打击各类违法犯罪，建设更高水平的平安中国、法治中国。（公安部牵头，年内持续推进）
　　十一、全面加强政府自身建设
　　（三十八）坚持不懈推进全面从严治党，加强法治政府、廉洁政府建设。

　　**46．**各级政府要全面贯彻落实党的十九大和十九届历次全会精神，深刻认识“两个确立”的决定性意义，增强“四个意识”、坚定“四个自信”、做到“两个维护”，自觉在思想上政治上行动上同以习近平同志为核心的党中央保持高度一致。依法接受同级人大及其常委会的监督，自觉接受人民政协的民主监督，主动接受社会和舆论监督。加强统计监督。支持工会、共青团、妇联等群团组织更好发挥作用。坚持不懈推进全面从严治党，深入开展党风廉政建设和反腐败斗争。加强廉洁政府建设。巩固党史学习教育成果。政府工作人员要自觉接受法律监督、监察监督和人民监督，始终把人民放在心中最高位置，无愧于人民公仆称号。（国务院各部门分别负责，年内持续推进）坚持依法行政，加强法治政府建设。（司法部牵头，有关部门按职责分工负责，年内持续推进）深化政务公开。（国务院办公厅牵头，年内持续推进）加强审计监督。（审计署牵头，年内持续推进）
　　（三十九）必须恪尽职守、勤政为民，凝心聚力抓发展、保民生。

　　**47．**坚持发展是第一要务，必须全面落实新发展理念，推动高质量发展。要锲而不舍落实中央八项规定精神，驰而不息纠治“四风”特别是形式主义、官僚主义，坚决反对敷衍应付、推诿扯皮，坚决纠治任性用权、工作方法简单粗暴。要始终把人民群众安危冷暖放在心上，察实情、办实事、求实效，及时回应民生关切，坚决严肃处理漠视群众合法权益的严重失职失责问题。要充分发挥中央和地方两个积极性，尊重人民群众首创精神，防止政策执行“一刀切”、层层加码，持续为基层减负。健全激励和保护机制，支持广大干部敢担当、善作为。全国上下毕力同心、苦干实干，就一定能创造新的发展业绩。（国务院各部门分别负责，年内持续推进）
　　十二、做好民族、宗教、侨务、国防、港澳台、外交工作
　　（四十）坚持和全面贯彻党的民族政策、宗教政策、侨务政策。

　　**48．**要坚持和完善民族区域自治制度，以铸牢中华民族共同体意识为主线，促进各民族交往交流交融，推动民族地区加快现代化建设步伐。（国家民委牵头，有关部门按职责分工负责，年内持续推进）坚持党的宗教工作基本方针，坚持我国宗教的中国化方向，积极引导宗教与社会主义社会相适应。（有关部门按职责分工负责，年内持续推进）全面贯彻党的侨务政策，维护海外侨胞和归侨侨眷合法权益，激励海内外中华儿女携手共创新的辉煌。（有关部门按职责分工负责，年内持续推进）
　　（四十一）大力支持国防和军队建设。

　　**49．**要深入贯彻习近平强军思想，贯彻新时代军事战略方针，扣牢建军一百年奋斗目标，全面加强党的领导和党的建设，全面深化练兵备战，坚定灵活开展军事斗争，捍卫国家主权、安全、发展利益。加快现代军事物流体系、军队现代资产管理体系建设，构建武器装备现代化管理体系，持续深化国防和军队改革，加强国防科技创新，深入实施新时代人才强军战略，推进依法治军、从严治军，推动军队高质量发展。加强全民国防教育。各级政府要大力支持国防和军队建设，深入开展“双拥”活动，让军政军民团结坚如磐石。（有关部门按职责分工负责，年内持续推进）优化国防科技工业布局。（国家国防科工局牵头，9月底前出台相关政策，年内持续推进）完成国防动员体制改革。（国家发展改革委等按职责分工负责，12月底前完成）
　　（四十二）继续全面准确、坚定不移贯彻“一国两制”、“港人治港”、“澳人治澳”、高度自治的方针。

　　**50．**落实中央对特别行政区全面管治权，坚定落实“爱国者治港”、“爱国者治澳”。全力支持特别行政区政府依法施政。支持港澳防控疫情、发展经济、改善民生，更好融入国家发展大局，保持香港、澳门长期繁荣稳定。（国务院港澳办牵头，有关部门按职责分工负责，年内持续推进）
　　（四十三）坚持对台工作大政方针。

　　**51．**贯彻新时代党解决台湾问题的总体方略，坚持一个中国原则和“九二共识”，推进两岸关系和平发展和祖国统一。坚决反对“***”分裂行径，坚决反对外部势力干涉。两岸同胞要和衷共济，共创民族复兴的光荣伟业。（有关部门按职责分工负责，年内持续推进）
　　（四十四）坚持独立自主的和平外交政策。

　　**52．**坚定不移走和平发展道路，推动建设新型国际关系，推动构建人类命运共同体。推进落实全球发展倡议，弘扬全人类共同价值。中国始终是世界和平的建设者、全球发展的贡献者、国际秩序的维护者，愿同国际社会一道，为促进世界和平稳定与发展繁荣作出新的更大贡献。（外交部、国家发展改革委、商务部、工业和信息化部、财政部、生态环境部、交通运输部、农业农村部、文化和旅游部、国家卫生健康委、国际发展合作署、国家乡村振兴局、国家疾控局等按职责分工负责，年内持续推进）
　　今年将召开中国共产党第二十次全国代表大会，是党和国家事业发展进程中十分重要的一年。我们要更加紧密地团结在以习近平同志为核心的党中央周围，高举中国特色社会主义伟大旗帜，以习近平新时代中国特色社会主义思想为指导，认真落实党中央、国务院决策部署，攻坚克难，砥砺奋进，努力完成全年目标任务，确保兑现对人民的承诺。一是强化组织领导。今年经济社会发展任务重、挑战多，各地区、各部门要进一步增强责任感、紧迫感，把责任扛在肩上，对各项工作早安排早部署，争取时间及早抓落实，细化时间表、路线图、优先序。有关地区和部门要对照分工意见，抓紧制定本地区、本部门落实重点工作分工的实施方案，于4月6日前报国务院。二是加强统筹协调。各地区、各部门要尊重发展规律、客观实际和群众需求，强化系统思维和科学谋划，力戒形式主义、官僚主义。要统筹稳增长、调结构、推改革，注重实际效果和可持续性，扎实做好稳中求进各项工作，脚踏实地为群众办实事、解难题，为市场主体减负纾困。三是密切协作配合。要加强跨部门、跨区域分工协作，形成工作合力。各部门要强化政策支撑和要素保障，加强调查研究，密切关注政策落实进展情况、困难问题和成效经验，根据形势变化调整完善政策。各地区要因地制宜创造性开展工作，有针对性地采取措施，确保政策落地见效。四是抓好监督落实。各地区、各部门要坚持言必信、行必果，以钉钉子精神坚韧不拔抓部署、抓落实、抓督查，有力有序推动各项重点工作如期完成，重大情况要及时报告。要规范督查督办，加大监督问责力度，防止和纠正推诿扯皮、敷衍塞责、“一刀切”特别是漠视群众合法权益受侵害等不作为乱作为，确保执行不偏向、不变通、不走样。国务院办公厅要建立《政府工作报告》督查总台账，定期对账督办，推动党中央、国务院重大决策部署不折不扣落到实处。

国务院
　　2022年3月21日

© 北大法宝：（[www.pkulaw.com](https://www.pkulaw.com/)) 专业提供法律信息、法学知识和法律软件领域各类解决方案。北大法宝为您提供丰富的参考资料，正式引用法规条文时请与标准文本核对。
欢迎查看所有[产品和服务](https://www.pkulaw.com/helps/2.html)。法宝快讯：[如何快速找到您需要的检索结果？](https://www.pkulaw.com/helps/70.html)[法宝](https://www.pkulaw.com/helps/69.html)V6有何新特色？

扫描二维码阅读原文

**原文链接：**<https://www.pkulaw.com/chl/8a9a7b1e80da46dabdfb.html>
